# Circulating Levels of CILP2 Are Elevated in Coronary Heart Disease and Associated with Atherosclerosis

**DOI:** 10.1155/2020/1871984

**Published:** 2020-10-31

**Authors:** Wenjing Hu, Ke Li, Hongdong Han, Shan Geng, Baoyong Zhou, Xiaoyun Fan, Shangcheng Xu, Mengliu Yang, Hua Liu, Gangyi Yang, Yongsheng Liu

**Affiliations:** ^1^Chongqing Prevention and Treatment Hospital for Occupational Diseases, 400000 Chongqing, China; ^2^Department of Endocrinology, The Second Affiliated Hospital, Chongqing Medical University, 400000 Chongqing, China; ^3^Department of Hepatobiliary Surgery, First Affiliated Hospital, Chongqing Medical University, 400000 Chongqing, China; ^4^School of Biomedical Sciences, The University of Queensland, 4702 Brisbane, Australia; ^5^Department of Pediatrics, University of Mississippi Medical Center, 32099, 2500 North State Street, 39200, Jackson, Mississippi, MS 39216-4505, USA

## Abstract

**Methods and Results:**

Circulating CILP2 levels (measured by ELISA) were compared to various insulin resistance- and atherosclerosis-related parameters in normal subjects and newly diagnosed CHD patients. THP-1 cells were cultured and treated with indicated stimulators. Western blots and RT-PCR were performed to examine protein and mRNA expressions. The results showed that there were significantly higher circulating CILP2 levels in CHD patients relative to healthy controls. Circulating CILP2 correlated positively with waist-hip ratio (WHR), total cholesterol (TC), low-density lipoprotein cholesterol (LDL-C), HbA1c, homeostasis model assessment of insulin resistance (HOMA-IR), and Gensini scores. In an *in vitro* study, we found that CILP2 increased oxidatively modified LDL-stimulated lipid accumulation in THP-1 macrophages *via* the upregulation of CD36 expression. Inhibition of PPAR*γ* signaling eliminated the CILP2 regulation of CD36 expression in THP-1 macrophages. CILP2 positively regulated CD36 transcription through PPAR*γ*-mediated action on two peroxisome-proliferator-responsive elements (PPREs) binding sites of CD36 promoter, PPRE-G, and PPRE-J.

**Conclusions:**

Our findings have uncovered a novel role for CILP2 in lipid uptake and foam cell formation. This role is mediated by CD36 through the activation of PPAR*γ* pathway.

## 1. Introduction

Coronary heart disease (CHD) is a complex disease caused by multiple factors, including heredity and environment. Recently, genome-wide association studies (GWAS) of different races have identified more than 30 loci associated with CHD [1]. One of these newly identified single nucleotide polymorphisms (SNPs) is rs16996148 SNP in the NCAN/cartilage intermediate layer protein 2 (CILP2)/PBX4. The rs16996148 is located on chromosome 19p13 in an intergenic region between CILP2 and PBX4 [2]. CILP2 may be a cartilage intermediate layer protein [2]. Recently, it has been revealed that rs16996148 of the *CILP2* gene is significantly correlated to high-density lipoprotein cholesterol (HDL-C) in an Asian Malay population [3]. More recently, rs16996148 has been reported to be associated with low-density lipoprotein cholesterol (LDL-C) and serum HDL-C concentrations but not associated with triglyceride (TG) concentrations in a Chinese population [4]. However, in several other GWAS, SNPs in proximity to CILP2 have been exhibited to be related to TG concentrations [5]. Therefore, the genetic variant of CILP2 may influence the risk of CHD through an effect on lipid levels in these populations.

CILP1 and CILP2 were initially found to be mainly expressed in cartilaginous tissues [6]. Subsequently, the CILP2 expression was also found in skeletal muscle and the myocardium. Many matrix molecules exist in skeletal muscle and the myocardium, including tenascin-C, decolorizer, and types I, III, V, and VI collagen [7–9]. Therefore, CILP2 may be related to the skeletal structure and matrix structures of connective tissue formation.

Recently, we reported that CILP2 was a secreted protein that existed in circulating blood in humans and animals [10]. We found that circulating CILP2 levels had a progressive increase from normal to impaired glucose tolerance (IGT) and then to diabetes, which was correlated with insulin resistance (IR) and obesity [10]. However, to our knowledge, there have been no reports showing the association between CILP2 and atherosclerosis in either humans or animals.

To further understand the relationship between CILP2 and cholesterol metabolism and atherosclerosis, we reported serum CILP2 concentrations in CHD patients and normal adults. Furthermore, we examined the possible role of CILP2 on atherosclerosis through both clinical studies and experiments *in vivo* and *in vitro*. Finally, we identified a direct interaction between CILP2 and CD36 in the regulation of cholesterol metabolism.

## 2. Materials and Methods

### 2.1. Cross-Sectional Studies

A total of 272 subjects, including 167 patients with newly diagnosed CHD (CHD group) and 105 healthy controls, were randomly recruited in this study from June 2016 to Dec 2017, by Hospital Management Information System (HIS). The diagnosis of CHD was confirmed by positive coronary angiography or angioplasty (angiographic evidence of at least one 50% diameter stenosis in one or more coronary arteries). The severity of CHD was assessed by the number of diseased vessels. Gensini score was determined by the number of stenosed coronary artery segments in CHD patients [11]. CHD subjects with concomitant valvular heart disease, acute and chronic viral or bacterial infections, acute renal failure, cardiomyopathy, diabetes, hypertension, or connective tissue diseases, and those undergoing coronary artery bypass surgery or on dialysis, were excluded from the study. Furthermore, acute coronary syndrome and heart failure patients were also eliminated. One hundred five age-matched healthy subjects without clinical evidence of major diseases were recruited from an unselected population that underwent a routine medical check-up and were used as the controls. These subjects had no family history of T2DM and CHD and were excluded CHD by CT coronary angiography (coronary CTA). Two study cohorts were shown in Figure S1. These subjects were not taking any medication. The study was conducted following the Declaration of Helsinki and was approved by the Human Research Ethics Committee of Chongqing Medical University (CHICTR- OPC-14005324). Written informed consent was obtained from each patient. The privacy rights of human subjects must always be observed.

### 2.2. Anthropometric Examine, Biochemical, and CILP2 Measurements

Body composition and anthropometry were measured in all patients and controls before breakfast. Body mass index (BMI) was calculated as weight (in kilograms) divided by height squared (in meters). Hip circumference and waist circumference (WC) were measured for the measurement of waist-to-hip ratio (WHR). The homeostasis model assessment of insulin resistance (HOMA-IR) was calculated using the following equations [12]: HOMA − IR = fasting insulin (FIns, *μ*U/mL) × fasting blood glucose (FBG, mmol/L)/22.5. Blood glucose and HbA1c were measured by the glucose-oxidase method or anion-exchange HPLC, respectively. Insulin and free fatty acids (FFA) were measured using the ELISA kit. Circulating CILP2 concentrations were determined using an ELISA Kit (Elisa Biotech Co., Ltd. Shanghai China). The limit of detection was 6.25 ng/L, and intra- and interassay variations were <8% and <10%, respectively. The linear range of the assay was 25-1600 ng/L [10]. Serum levels of blood fat, including HDL-C, LDL-C, TG, and total cholesterol (TC), were analyzed enzymatically using an auto-analyzer.

### 2.3. Animals

Male C57BL/6J and ApoE KO mice (28 to 30g) were purchased from the animal center at the Chongqing Medical University. Animals were maintained in a temperature-controlled room with a 12-hour light/dark cycle and free access to water and a standard chow diet (SD) until they were 18 weeks old. Eight-week-old male ApoE KO mice were divided into two groups (*n* = 6 for each group) and fed with an SD or high-fat diet (HFD) for 12 weeks. Mice were sacrificed by Metofane overdosing inhalation (5%), and the aorta samples of mice were immediately shock-frozen and stored at -160°C until used for RNA or protein extractions. Serum samples were stored at –80°C for later analysis. All experiments were approved by the Ethics Review Committee for Animal Experimentation of Chongqing Medical University. Formal permission to generate and use genetically modified animals was obtained from the responsible local authorities (permit number SCXK 2018-0003). This study conformed to the guidelines from Directive 2010/63/EU of the European Parliament on the protection of animals used for scientific purposes or the NIH guidelines.

### 2.4. Recombinant Adenovirus Vectors

Adenoviral vectors expressing CILP2 (Ad-*CILP2*) or green fluorescent protein (Ad-*GFP*) were constructed using the AdEasy Adenoviral Vector System (Qbiogene), as previously described [13], and the potency of expression was evaluated *in vitro*. Large-scale amplification and purification of adenoviruses were performed with the ViraBind Adenovirus Purification Kit (Cell Biolabs, San Diego, USA), and recombinant adenoviruses were stored at -80°C.

### 2.5. Cell Culture Experiments

Human THP-1 cells (American Type Culture Collection, Manassas, VA, USA) were cultured in RPMI-1640 with 10% fetal calf serum (FBS; Invitrogen) in 6-well trays and treated with oxidatively modified LDL (oxLDL) at various concentrations (0, 1, 5, 10, 20, and 50 *μ*g/mL) for 24 h or incubated with oxLDL (20 *μ*g/mL) for indicated times (0–48 h). For GILP2 overexpression experiments, THP-1 cells were transfected with Ad-*CILP2* or Ad*-GFP* for 48 h and then incubated with oxLDL for 24 h. For signal pathway experiments, GW9662, a peroxisome proliferator-activated receptor-*γ* (PPAR*γ*) antagonist (20 *μ*mol/L; Sigma) or PPAR*γ* agonist rosiglitazone (20 *μ*mol/L; Cayman Chemical), was added to cell cultures 60 min before adenovirus transfection. THP-1 cells were differentiated into macrophages by incubation for 24 h with phorbol myristate acetate (PMA; 320nM, Sigma) before all experiments. For cell apoptosis measurement, THP-1 cells were incubated with oxLDL for 24 h, 48 h, and 72 h, and then, cell apoptosis was analyzed by flow cytometry (FACS Vantage SE, BD, Franklin Lakes, NJ, USA).

### 2.6. Lipid Staining by Oil Red O

THP-1 cells were pretreated with Ad-*CILP2* or Ad-*GFP* for 48 h, and cultured with oxLDL (20 *μ*g/mL) for 24 h, and then washed three times with PBS. Cells were fixed with 4% formaldehyde for 30 min and then stained with Oil Red O for 60 min at 25°C.

### 2.7. Immunohistochemistry (IHC) Staining

IHC was performed following the standard protocol described previously [14]. The slides were blocked with the primary antibody and anti-CILP2 and incubated at 4°C overnight. Then, goat anti-rabbit immunoglobulin was used on the section and incubated for 30 min at 37°C. Finally, sections were developed with diaminobenzidine.

### 2.8. Luciferase Assays

The human CD36 promoter [*pGL3-CD36 (-404/+187)*] was synthesized from human genomic DNA. CD36 other reporter genes, including *pGL3-CD36 (-311/+187)-luc*, *pGL3-CD36 (-225/+187)-luc*, and *pGL3-CD36 (-102/+187)-luc*, were also prepared by PCR to generate various segments of the sequences between *-404* and *+187* bp in CD36 gene promoter (Table S1). All fragments were cloned into the pGL3-luciferase plasmid (TIANGEN, Beijing, China) and sequenced to confirm orientation and sequence. Transient transfections were performed on THP-1 cells. After 24 hours of transfection, luciferase activities were assayed [15].

### 2.9. Protein and mRNA Analysis

Real-time quantitative PCR (RT-PCR) was performed as reported previously [16]. The primer pairs used for RT-PCR were listed in Table S2. Protein analysis was performed with Western blotting, as described previously [16]. Primary antibodies included anti-CILP2, anticlass B scavenger receptor (CD36) (Abcam, Cambridge, MA, USA), and anti-*β*-actin (Santa Cruz Biotechnology, Inc. Dallas, TX, USA).

### 2.10. Statistical Analysis

Statistical Package for Social Sciences version 15.0 (SPSS, Chicago, IL) was used for all analyses. Data were mean ± SD or median (interquartile). Comparisons between groups were performed by ANOVA, unpaired *t*-test, or paired *t*-test. The normal distribution of the data was tested using the Kolmogorox-Smirnov test. Simple and multiple linear stepwise regression analyses were used to examine the association between circulating CILP2 and the values of other biomarkers. Odds ratios (OR) were calculated for an increase of each quartile of CILP2 in subgroups of the strata variables. In all statistical tests, *p* values less than 0.05 were considered significant. Sample size was calculated using the following equations: *n* = (*Z*_*α*/2_*σ*/*εμ*)^2^, where *σ* is the standard, *μ* is the mean, Z_*α*/2_ = 1.96, *α* = 0.05, and *ε* = 6%.

## 3. Results

### 3.1. Serum CILP2 Concentration and Its Association with Other Parameters in Healthy or CHD Subjects

**Figure 1:**
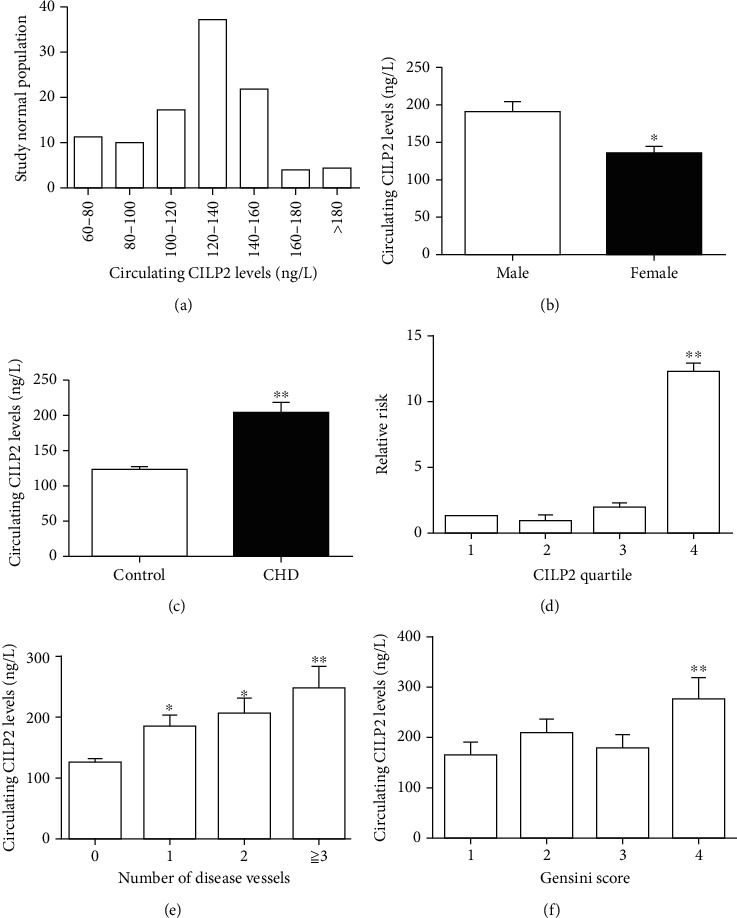
Circulating CILP2 levels in the study population. (a) Distribution of CILP2 concentrations in healthy individuals (*n* = 105). (b) Circulating CILP2 levels according to sex (*n* = 157 for male, *n* = 115 for female). (c) Circulating CILP2 levels in normal and CHD subjects (*n* = 105 for controls, and *n* = 167 for CHD). (d) Prevalence of increased CHD in different quartiles of CILP2: quartile 1 (*n* = 68), < 104.7 ng/L; quartile 2 (*n* = 67), 104.7-133.7 ng/L; quartile 3 (*n* = 69), 133.7-163.3 ng/L; quartile 4 (*n* = 68), > 163.3 ng/L. (e) Circulating CILP2 concentrations in CHD patients according to the number of stenotic coronary arteries (number 0, *n* = 105; number 1, *n* = 78; number 2, *n* = 46; number 3, *n* = 43). (f) Increased CILP2 levels in different quartiles of Gensini score: quartile 1 (*n* = 43), < 12; quartile 2 (*n* = 41), 12-20; quartile 3 (*n* = 42), 20-42; quartile 4 (*n* = 41), > 42. Data were means ± SD, ^∗^*p* < 0.05 or ^∗∗^*p* < 0.01 vs. male, controls, quartile 1, or number 0.

The clinical characteristics of the participants in this study were summarized in Table 1, and no missing data were found in this experiment. There were no marked differences in BMI, age, FFA, HbA1c, diastolic blood pressure (DBP), and TC between controls and CHD patients. However, those with CHD had higher WHR, SBP, TG, LDL-C, FBG, 2 h postglucose load blood glucose (2h-BG), FIns, HOMA-IR, Gensini score, and lower HDL-C levels than healthy adults. We measured circulating CILP2 concentrations in 105 healthy individuals after overnight fasting. Circulating CILP2 levels displayed skewed distribution (Figure 1(a)). As shown in Figure 1(b), circulating CILP2 levels were significantly higher in men (140.2 (123.3-226.6) ng/L) than women (128.4 (97.4-148.7) ng/L; *p* < 0.05). Importantly, the concentrations of circulating CILP2 were significantly higher in CHD patients than those in normal subjects (*p* < 0.01, Figure 1(c)). Next, we investigated the relationship between serum CILP2 and other parameters in CHD patients using linear correlation. Circulating CILP2 positively correlated with HbA1c, TC, LDL-C, WHR, HOMA-IR, and Gensini score, whereas negatively correlated with HDL-C (Table 2). All these correlations persisted after adjusting for age. Finally, to determine variables independently associated with circulating CILP2, a multiple stepwise regression was performed. Our data revealed that WHR, LDL-C, and Gensini score were independently associated with CILP2 (Table 2). The equation of multiple regression is as follows: *Y*_CILP2_ = 0.985 + 1.087*X*_WHR_ + 0.067X_LDL_ + 0.020X_Gensini score_ (*R*2 = 0.351, *p* < 0.01). In addition, the elevation of the CILP2 level displayed a linear trend and was independent related to CHD. Cochran-Armitage trend test and row mean score difference were also performed to analyze CILP2 concentrations (Table S3). With the increase of the CILP2 quartile, the relative risk of CHD increased significantly (Figure 1(d)). When the study population was divided into four groups by the number of damaged vessels (*N* = 0, 1, 2, ≥3), circulating CILP2 levels in the group *N* = 1, 2, and ≥3 were significantly higher than those in group *N* = 0 (*p* < 0.05 and *p* < 0.01, Figure 1(e)). Finally, when subjects were stratified according to Gensini score, a significant increase of CILP2 level was observed in the highest quartile of Gensini score related to the lowest one (*p* < 0.01, Figure 1(f)).

### 3.2. CILP2 Expression in the Aorta of Mice and oxLDL-Stimulated Macrophages

**Figure 2:**
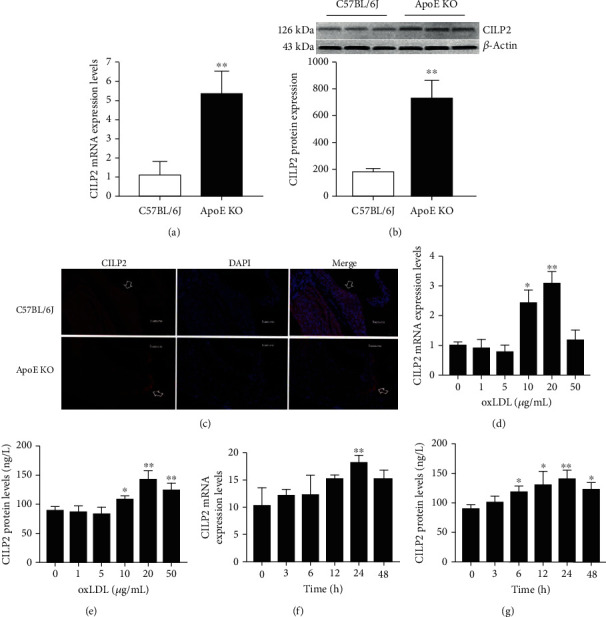
CILP2 expression in the aorta of mice and oxLDL-stimulated THP-1 macrophages. CILP2 mRNA (a) and protein (b) expression in the aorta of C57BL/6J and ApoE KO mice (*n* = 3‐5). (c) IHC staining for the CILP2 expression in the aortic section of C57BL/6J and ApoE KO mice. (d, e) THP-1 macrophages incubated with oxLDL at various concentrations for 24 h. (d) CILP2 mRNA expression in cell lysates. (e) CILP2 protein levels in the culture medium. (f, g) THP-1 macrophages incubated with oxLDL (20 *μ*g/mL) for indicated times (0–48 h). (f) CILP2 mRNA expression in cell lysates. (g) CILP2 protein levels in the culture medium. Data were expressed as mean ± SD. ^∗^*p* < 0.05 and ^∗∗^*p* < 0.01 vs. C57BL/6J, 0 *μ*g/mL, or 0 h.

To investigate whether CILP2 was associated with atherosclerosis, we examined the CILP2 expression in the aorta samples and circulating CILP2 levels in ApoE KO mice, a well-characterized animal model of atherosclerosis. Similar to the levels of circulating CILP2 in CHD patients, the mRNA and protein levels of CILP2 in the aorta samples of ApoE KO mice were significantly elevated as compared with control mice (Figures 2(a) and 2(b)). IHC staining showed that the CILP2 expression was significantly increased in the aortic section of ApoE KO mice than that in WT mice (Figure 2(c)). In addition, circulating CILP2 levels were significantly increased in ApoE KO mice compared with WT mice (Fig. S2). In HFD-fed ApoE KO mice, circulating CILP2 levels were higher than in SD-fed ApoE KO mice (Figure S3). These data further suggest that CILP2 may be associated with atherosclerosis.

Intimal macrophages uptake oxLDL and form foam cells. This process plays a key role in the occurrence of atherosclerosis. To investigate the effect of foam cell formation on the CILP2 expression, we first examined whether CILP2 was expressed in macrophages. As shown in Fig. S4A, the expression of CILP2 protein was found in the cultured THP-1 macrophages. To further reveal the ability of oxLDL to stimulate the CILP2 expression in THP-1 cells, we examined whether oxLDL regulated CILP2 mRNA expression in these macrophages. We first found that THP-1 cell apoptosis was increased after incubation with oxLDL (Fig. S5). In addition, the results showed a time- and dose-dependent pattern for the oxLDL-induced CILP2 mRNA expression (Figures 2(d)–2(g)). The most prominent effect of oxLDL on CILP2 mRNA was found at 20 *μ*g/mL of oxLDL incubation for 24 h. CILP2 protein levels in culture medium showed a similar change (Figures 2(e) and 2(g)). This concentration was comparable to circulating oxLDL concentrations in CHD individuals [15].

### 3.3. CILP2 Promotes Lipid Uptake and the Formation of Foam Cell In Vitro

**Figure 3:**
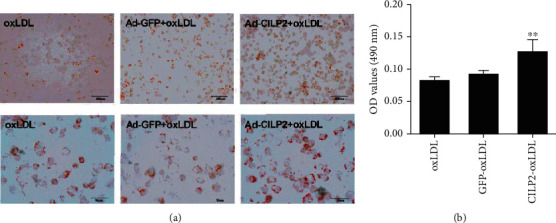
Effect of CILP2 on foam cell formation in THP-1 macrophages. (a) THP-1 cells were transfected with Ad-*GFP* or Ad-*CILP2* for 48 h and then incubated with oxLDL for 24 hours. Cells were stained with Oil Red O. (b) Quantitative analysis of Oil Red O staining. Data are expressed as mean ± SME. ^∗∗^*p* < 0.01 vs. GFP-oxLDL.

To evaluate the impact of CILP2 on forming foam macrophages, THP-1 macrophages were pretreated with Ad-*CILP2* or Ad-*GFP* and/or then incubated with oxLDL. As shown in Figure S4B-D, THP-1 macrophages were transfected successfully by Ad-*CILP2*, and the CILP2 expression was markedly evaluated at both mRNA and protein levels. In Ad-*CILP2*-infected THP-1 macrophages, Oil Red O staining demonstrated an increase in the number of intracellular lipid droplets compared with Ad-*GFP*-treated cells (Figures 3(a) and 3(b)). These data demonstrated that CILP2 induced stimulatory effects on lipid uptake to form foam cells in oxLDL stimulated macrophages.

### 3.4. CILP2 Increases CD36 and LOX-1 Expression in THP-1 Cells

**Figure 4:**
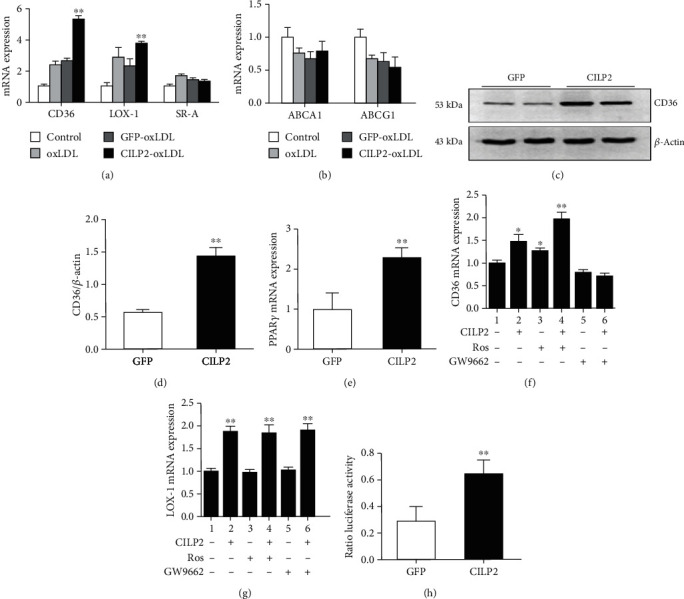
CILP2 modulates the CD36 expressions *via* the PPAR*γ* signaling pathway. THP-1 macrophages were treated as in the Methods. (a) CD36, LOX-1, and SR-A mRNA expression. (b) ABCA1 and ABCG1 mRNA expression. (c) CD36 protein levels. (d) Quantitative analysis of CD36 protein levels. (e) PPAR*γ* mRNA expression. (f, g) Effects of GW9662 and rosiglitazone treatment on CD36 (f) and LOX-1 (g) mRNA expression. (h) CILP2-mediated transcriptional activation in the full-length CD36 promoter. Data are expressed as mean ± SME. ^∗^*p* < 0.05 and ^∗∗^*p* < 0.01 vs. GFP-oxLDL or GFP or lane 1.

To elucidate the potential pathogenic role of increasing the CILP2 level *in vivo and in vitro*, we transfected THP-1 macrophages with Ad-*CILP2* or Ad-*GFP* in the presence of Dil-oxLDL. We then measured the ability of CILP2 to regulate CD36, lectin-like oxidized low-density lipoprotein receptor-1(LOX-1), and class A scavenger receptor (SR-A) in THP-1 macrophages. Overexpression of CILP2 significantly enhanced the mRNA levels of CD36 and LOX-1 (Figure 4(a)), but SR-A, ABCA (ATP-binding cassette transporter subfamily A member 1), and ABCG1 were unchanged (Figure 4(b)). In addition, the protein levels of CD36 were also significantly increased in Ad-*CILP2*-treated cells (Figures 4(c) and 4(d)). Our data further suggest the pathogenic relevance between CILP2 and atherosclerosis.

### 3.5. CILP2 Modulates LOX-1 and CD36 Expression through a PPAR*γ* Signaling Pathway

To understand the mechanism of the CILP2 regulation of the LOX-1 and CD36 expression, we investigated the effect of CILP2 on the PPAR*γ* expression in macrophages. The results showed that PPAR*γ* mRNA levels were markedly increased in Ad-*CILP2*-infected macrophages compared with those of the controls (Figure 4(e)). Next, we examined the ability of a specific PPAR*γ* inhibitor (GW9662) and agonist (rosiglitazone, Ros) to modulate the impacts of CILP2 on LOX-1 and CD36. When THP-1 cells were exposed to Ros, CILP2 treatment further increased the CD36 expression (Figure 4(f)), but the LOX-1 expression was unchanged (Figure 4(g)). Importantly, when THP-1 cells were treated by GW9662, the CILP2's effect on the CD36 expression was completely abolished (Figure 4(f)), whereas the LOX-1 expression was unchanged (Figure 4(g)). The results indicated that the PPAR*γ* signal was involved in the CILP2 regulation of the CD36 expression but not the LOX-1 expression.

### 3.6. The CILP2 Regulation of CD36 Transcription Is Mediated by PPAR*γ* through Two PPREs Binding Sites

**Figure 5:**
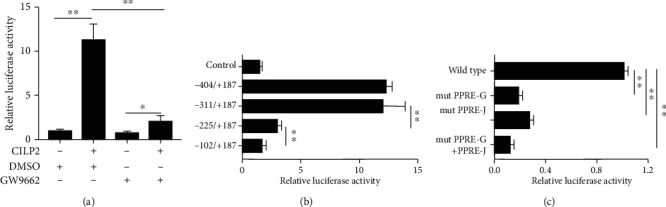
Transcriptional regulation of CD36 promoter by CILP2 *via* PPAR*γ* pathway. (a) CILP2-mediated transcription of CD36 promoter was blocked by GW9662 in THP-1 cells. (b) Deletion analysis of CD36 promoter. THP-1 cells were transfected with CILP2 overexpression vector, together with a series of truncated CD36 promoter-driven luciferase reporters. 48 h after the transfection, the luciferase activity was measured and expressed in relative luciferase units. (c) Site-directed mutagenesis analysis. THP-1 cells were cotransfected with the CILP2 expression and luciferase reporter plasmids containing WT and PPRE-G or PPRE-J binding site mutant CD36 promoters, and luciferase activity was measured. Data are expressed as the mean ± SE. ^∗∗^*p* < 0.01.

To investigate the molecular basis for the CILP2 regulation of CD36 transcription, we identified a potential CILP2 binding site in the CD36 promoter in THP-1 cells. As shown in Figure 4(h), in accordance with the regulation of the CD36 expression, we found that CILP2 increased the transcriptional activity of CD36 promoter. However, CILP2-induced CD36 transcriptional activity was blocked by GW9662 (Figure 5(a)), further suggesting that CILP2 regulation of CD36 transcription is mediated by PPAR*γ*.

To further determine the binding site of CILP2 on CD36 promoter, the human CD36 promoter from *-404* to*+187* bp were analyzed in THP-1 cells. For a series of truncated human CD36 promoters, luciferase activity assays showed that the deletion of the *-404/-225* bp region had no effect on CILP2-induced CD36 transcription, but deletion of the *-225/+187 bp* region including the peroxisome-proliferator-responsive elements (PPREs) site blocked the transcriptional activity of CD36 promoter (Figure 5(b)). Site-directed mutation in two PPREs binding sites (*-120* to *108* bp for PPRE-J and *-245* bp to *-233* bp for PPRE-G,) resulted in the significant decrease of CILP2-induced CD36 transcriptional activity (a decrease of 83.5% for PPRE-G and 71.8% for PPRE-J) (Figure 5(c)). These results showed that PPRE-G and PPRE-J binding sites were necessary for the CILP2 regulation of CD36 promoter activity.

## 4. Discussion

In this paper, we presented the first human study for CHD on circulating CILP2 levels using a highly specific ELISA. In addition, it was noteworthy that CILP2 levels in men were higher than those in female subjects. This protein may have sex-specific activity or regulation, or the discrepancy may be caused by other factors, such as lower fat content in men. However, importantly, we have also shown that serum CILP2 concentration was significantly higher in newly diagnosed patients with CHD, suggesting that CILP2 might be associated with atherosclerosis. Furthermore, CILP2 was markedly correlated with the parameters of obesity (WHR), IR (HOMA-IR), glucose metabolism (HbA1c), and atherosclerosis (TC, LDL-C, HDL-C, and Gensini score) in our study population. Multiple stepwise regressions showed that WHR, LDL-C, and Gensini score were independently related factors with circulating CILP2. The relative risks for CHD and the number of diseasing vessels were significantly elevated along with increasing CILP2 quartiles. These findings support the view that CILP2 is related to atherosclerosis, dyslipidemia, and obesity-related IR. However, our study has also some limitations. The cross-sectional design of this study leads to our inability to deduce a causal relationship between circulating CILP2 and CHD. Prospective studies are needed to clarify their precise interrelationship.

To further study the pathophysiological role of CILP2 *in vivo* and *in vitro*, we performed in-depth studies at the animal and molecular biological levels. Similar to the observation in humans, the mRNA and protein levels of CILP2 in the aorta samples of ApoE KO mice were significantly higher than in those of control mice, further confirming that CILP2 is related to atherosclerosis. Therefore, we propose that elevated circulating CILP2 in patients with CHD may be a defensive response against metabolic stress, such as dyslipidemia, or resistance to atherosclerosis. Although these results cannot prove causality between CILP2 and atherosclerosis, credible assumptions can be made and confirmed by future prospective cohort and mechanism studies.

One of the early stages of atherosclerosis is the accumulation of oxLDL in the intima and then the uptake of lipoproteins by macrophages, leading to the formation of foam cells [17]. In the present study, we found that the CILP2 expression was upregulated in a time- and dose-dependent manner in oxLDL-stimulated THP-1 cells. Therefore, we consider that the increased CILP2 expression in oxLDL-treated macrophages may be associated with an increase in oxLDL uptake. Furthermore, it is also interesting to investigate whether upregulation of scavenger receptors (SR) occurs in CILP2-stimulated THP-1 macrophages, and if so, it can be partly explained that in CHD patients, increased circulating CILP2 is associated with increased oxLDL uptake in macrophages. Therefore, we screened THP-1 cells for arteriosclerosis-related genes after transfection with pAd-*CILP2*. A major finding was the noticeable upregulation of LOX-1 and CD36, and this upregulation was accompanied by increased oxLDL uptake and the formation of foam cells in macrophages.

Macrophages can devour chemically modified LDLs, like acetylated LDL (acetyl LDL) and oxLDL, through SR, and convert them to form foam cells *in vitro* [17]. SRs are a group of transmembrane proteins involved in cell functions, such as adhesion and removal of apoptotic cells and modified lipoproteins. Several identified SRs including SR-A [18], class B (CD36) [19], and LOX-1 [20]. SR-A, (class A), LOX-1(class E), and CD36 (class B) are account for about 90% of oxLDL uptake [21, 22]. Therefore, these SRs exert a key role in the pathogenesis of atherosclerosis by recognizing and promoting the uptake of oxLDL by macrophages [21, 23]. The changes in the SR expression in human atherosclerosis have been reported in some studies [24, 25].

In this study, we found that CILP2 might be a circulating biomarker of two SRs, CD36 and LOX-1, suggesting a new role for CILP2 in mediating foam cell formation. However, CILP2 did not affect the SR-A, ABCA1, and ABCG1 expressions in THP-1 macrophages. It has been well known that reverse cholesterol transport is mediated by ABCG1 and ABCA1, two key mediators of cholesterol efflux, which are responsible for pumping cholesterol out of macrophages onto HDL particles [26]. Therefore, our results revealed that CILP2 failed to regulate cholesterol outflow but promoted oxLDL uptake mediated by CD36. Given the fact that CD36 is associated with the formation of foam cell and atherosclerosis [27, 28], overexpression of CD36 and the increase of oxLDL uptake mediated by CILP2 are likely promoting atherosclerosis.

Previous studies have found that PPARs play an important role in the regulation of SRs, lipid accumulation, and inflammatory response in macrophages [29]. In the PPARs family, macrophages mainly express PPAR gamma (PPAR*γ*) [30]. Previous reports revealed that orphan nuclear receptors such as PPAR*γ* and liver X receptors (LXRs) acted on a common target CD36 [31]. Therefore, we were highly concerned about whether CILP2 regulates CD36 mediated by PPAR*γ*. Therefore, we further studied the possible interaction between serum CILP2 and PPAR*γ* in THP-1 cells. Firstly, we observed that overexpression of CILP2 in macrophages led to an increase of the PPAR*γ* expression. Importantly, we found an elevated role of rosiglitazone, a PPAR*γ* agonist, and a lowering impact of GW9662, a PPAR*γ* antagonist, on the CILP2-stimulated CD36 expression. Based on these findings, we were confident that PPAR*γ* signaling was required for the CILP2-induced expression of CD36.

To explore the exact molecular mechanism by which CILP2 promotes the CD36 expression, we further found that CILP2 regulated the transcription of CD36 promoter in THP-1 macrophages by double luciferase assays. Furthermore, a PPAR*γ* inhibitor, GW9662, completely blocked the response of CD36 promoter to CILP2, suggesting a mechanism by which the role of CILP2 on CD36 promoter is mediated by PPAR*γ*. Although several lines of evidence indicated the relationship between PPAR*γ* and CD36 [32–34], our data unveiled a completely new role of CILP2 regulation for the transcriptional activity of CD36. Finally, the truncated and site-directed mutation analysis of CD36 promoter showed that CILP2 positively regulated CD36 transcription through PPAR*γ*-mediated action on two PPREs binding sites of CD36 promoter, PPRE-G, and PPRE-J.

In summary, the current study demonstrated that circulating concentrations of CILP2 were markedly increased in newly diagnosed CHD patients and closely related to the severity of CHD. We also uncovered a novel role for CILP2 in lipid uptake and foam cell formation. This role was mediated by CD36 through the activation of the PPAR*γ* pathway.

## Figures and Tables

**Table 1:** Clinical characteristics and circulating CILP2 levels of study subjects.

Variable	Controls (*n* = 105)	CHD (*n* = 167)	*p*
Males/female	59/46	98/69	0.587
Age (yr)	61.6 ± 9.8	62.6 ± 9.4	0.111
BMI (kg/m^2^)	23.2 ± 3.0	23.4 ± 3.3	0.704
WHR	0.87 ± 0.06	0.93 ± 0.06	< 0.001
SBP (mmHg)	118.9 ± 17.0	128.7 ± 17.5	< 0.001
DBP (mmHg)	73.9 ± 10.4	75.8 ± 9.9	0.147
TG (mmol/L)	1.22 (0.88,174)	1.31 (0.96,1.98)	0.029
TC (mmol/L)	4.60 ± 1.05	4.84 ± 1.19	0.886
HDL-C (mmol/L)	1.34 ± 0.30	1.14 ± 0.30	< 0.001
LDL-C (mmol/L)	2.42 ± 0.64	2.66 ± 0.95	< 0.05
FFA (*μ*mol/L)	0.53 (0.39,0.69)	0.55 (0.42,0.71)	0.405
FBG (mmol/L)	5.02 ± 0.62	5.32 ± 0.46	< 0.001
2 h-BG (mmol/L)	6.08 ± 1.16	6.71 ± 0.83	< 0.001
FIns (mU/L)	7.26 ± 3.49	14.84 ± 9.69	< 0.001
HbA1c (%)	5.75 ± 0.57	5.65 ± 0.33	0.077
HOMA-IR	1.64 ± 0.82	3.56 ± 2.26	< 0.001
CILP2 (ng/L)	126.9 (98.0, 141.7)	205.6 (122.0, 411.9)	< 0.001
Gensini score	—	18.0 (11.5-34.0)	< 0.001

BMI: body mass index; SBP: systolic blood pressure; DBP: diastolic blood pressure; WHR: waist-to-hip ratio; FBG: fasting blood glucose; 2 h-BG: 2 h postglucose load blood glucose; FIns: fasting plasma insulin; HOMA-IR: HOMA-insulin resistance index; FFA: free fatty acids; TG: triglyceride; TC: total cholesterol; HDL-C: high-density lipoprotein cholesterol; LDL-C: low-density lipoprotein cholesterol; HbA1c: glycosylated hemoglobin; CILP2: cartilage intermediate layer protein 2. Data are mean ± SD or median (interquartile).

**Table 2:** Linear and multiple regression analysis of variables associated with circulating CILP2^a^ levels in CAD patients.

Variable	Simple		Multiple	
	*R*	*p*	*b*	*p*
Age (yr)	0.032	0.608	—	—
BMI (kg/m^2^)	-0.063	0.307	—	—
WHR	0.412	< 0.001	1.087	< 0.001
SBP (mmHg)	-0.027	0.663	—	—
DBP (mmHg)	-0.114	0.063	—	—
TG (mmol/L)^a^	-0.011	0.854	—	—
TC (mmol/L)	0.129	< 0.05	—	—
HDL-C (mmol/L)	-0.220	< 0.001	—	—
LDL-C (mmol/L)	0.230	< 0.001	0.067	< 0.001
FFA (*μ*mol/L)^a^	-0.016	0.155	—	—
HbA1c (%)	0.204	< 0.05	—	—
HOMA-IR	0.184	< 0.05	—	—
Gensini score^a^	0.383	< 0.001	0.020	< 0.001

^a^Log transformed before analysis. In multiple linear stepwise regression analysis, values included for analysis were age, BMI, WHR, SBP, DBP, TG, TC, HDL-C, LDL-C, FFA, HbA1c, HOMA-IR, and Gensini score.

## Data Availability

The data that support the findings of this study are available from the corresponding author upon reasonable request.

## Abbreviations

GWAS: Genome-wide association studies
CHD: Coronary heart disease
WHR: Waist-hip ratio
TC: Total cholesterol
LDL-C: Low-density lipoprotein cholesterols
HOMA-IR: Homeostasis model assessment of insulin resistance
oxLDL: Oxidatively modified LDL
LOX-1: Lectin-like oxidized LDL receptor-1
CD36: Cluster of differentiation 36
SR-A: Class A scavenger receptor
PPAR*γ*: The peroxisome proliferator-activated receptor (PPAR)-*γ*
PPREs: Peroxisome-proliferator-responsive elements
SNPs: Single nucleotide polymorphisms
IGT: Impaired glucose tolerance
IR: Insulin resistance
BMI: Body mass index
WC: Waist circumference
FBG: Fasting blood glucose
FFA: Free fatty acids
TC: Total cholesterol
TG: Triglyceride
OR: Odds ratios
2 h-BG: 2 h postglucose load blood glucose
ABCA: ATP-binding cassette transporter subfamily A member
SR: Scavenger receptors.

## Supplementary Materials

Supplementary Materials See Supplementary Figures 1-5 and Supplementary Tables 1–3 in the Supplementary Material for comprehensive image analysis. Supplementary Table 1: the specific sequences used for CD36 promoter recombinant plasmids and site-directed mutation. Supplementary Table 2: the specific primers sequence used for RT-qPCR analysis. Supplementary Table 3: row mean scores and Cochran–Armitage trend test of the impact of circulating CILP2 on CHD patients. Supplementary Figure 1: flow of the study. Supplementary Figure 2: circulating CILP2 levels in SD-fed WT and ApoE KO mice (*n* = 6 for each group). Data are shown as mean ± SD. ^∗∗^*p* < 0.01 vs.C57BL/6J. Supplementary Figure 3: circulating CILP2 levels in SD- or HFD-fed ApoE KO mice. Eight-week-old male ApoE KO mice were divided into two groups (*n* = 6 for each group) and fed with a SD or HFD for 12 weeks. Data were shown as mean ± SD. ^∗^*p* < 0.05 vs. prefeeding. Supplementary Figure 4: the expression of CILP2 protein was found in the cultured THP-1 macrophages (A), THP-1 macrophages were transfected successfully by Ad-*CILP2*, and CILP2 expression was markedly evaluated at both mRNA and protein (B-D). Data are shown as mean ± SEM. ^∗∗^*p* < 0.01 vs. GFP. Supplementary Figure 5: cell apoptosis was determined by flow cytometry assay. Data were shown as mean ± SEM. ^∗∗^*p* < 0.01 vs. 0 h.

## References

[1] Lee J. Y., Lee B. S., Shin D. J. (2013). A genome-wide association study of a coronary artery disease risk variant. *Journal of Human Genetics*.

[2] Johnson K., Farley D., Hu S. I., Terkeltaub R. (2003). One of two chondrocyte-expressed isoforms of cartilage intermediate-layer protein functions as an insulin-like growth factor 1 antagonist. *Arthritis and Rheumatism*.

[3] Tai E. S., Sim X. L., Ong T. H. (2009). Polymorphisms at newly identified lipid-associated loci are associated with blood lipids and cardiovascular disease in an Asian Malay population. *Journal of Lipid Research*.

[4] Zhou L., Ding H., Zhang X. (2011). Genetic variants at newly identified lipid loci are associated with coronary heart disease in a Chinese Han population. *PLoS One*.

[5] Jeemon P., Pettigrew K., Sainsbury C., Prabhakaran D., Padmanabhan S. (2011). Implications of discoveries from genome-wide association studies in current cardiovascular practice. *World Journal of Cardiology*.

[6] Eyre D. (2002). Collagen of articular cartilage. *Arthritis Research*.

[7] Light N., Champion A. E. (1984). Characterization of muscle epimysium, perimysium and endomysium collagens. *The Biochemical Journal*.

[8] Listrat A., Picard B., Geay Y. (1999). Age-related changes and location of type I, III, IV, V and VI collagens during development of four foetal skeletal muscles of double- muscled and normal bovine animals. *Tissue & Cell*.

[9] Bock H. C., Michaeli P., Bode C. (2001). The small proteoglycans decorin and biglycan in human articular cartilage of late-stage osteoarthritis. *Osteoarthritis and Cartilage*.

[10] Wu T., Zhang Q., Wu S. (2019). CILP-2 is a novel secreted protein and associated with insulin resistance. *Journal of Molecular Cell Biology*.

[11] Gensini G. G. (1983). A more meaningful scoring system for determining the severity of coronary heart disease. *The American Journal of Cardiology*.

[12] Albareda M., Rodríguez-Espinosa J., Murugo M., de Leiva A., Corcoy R. (2000). Assessment of insulin sensitivity and beta-cell function from measurements in the fasting state and during an oral glucose tolerance test. *Diabetologia*.

[13] Yang M., Dai J., Jia Y. (2014). Overexpression of juxtaposed with another zinc finger gene 1 reduces proinflammatory cytokine release via inhibition of stress-activated protein kinases and nuclear factor-*κ*B. *The FEBS Journal*.

[14] Zhang C., Luo X., Chen J. (2019). Osteoprotegerin promotes liver steatosis by targeting the ERK–PPAR-*γ*–CD36 pathway. *Diabetes*.

[15] Zhou J., Zhai Y., Mu Y. (2006). A novel pregnane X receptor-mediated and sterol regulatory element-binding protein-independent lipogenic pathway. *The Journal of Biological Chemistry*.

[16] Yuan L., Luo X., Zeng M. (2015). Transcription factor TIP27 regulates glucose homeostasis and insulin sensitivity in a PI3-kinase/Akt-dependent manner in mice. *International Journal of Obesity*.

[17] Holvoet P., Vanhaecke J., Janssens S., van de Werf F., Collen D́́. (1998). Oxidized LDL and malondialdehyde- modified LDL in patients with acute coronary syndromes and stable coronary artery disease. *Circulation*.

[18] Boullier A., Bird D. A., Chang M.-k. (2001). Scavenger receptors, oxidized LDL, and atherosclerosis. *Annals of the New York Academy of Sciences*.

[19] Epstein F. H., Steinberg D., Parthasarathy S., Carew T. E., Khoo J. C., Witztum J. L. (1989). Beyond cholesterol. *New England Journal of Medicine*.

[20] Palinski W., Rosenfeld M. E., Yla-Herttuala S. (1989). Low density lipoprotein undergoes oxidative modification in vivo. *Proceedings of the National Academy of Sciences*.

[21] Kodama T., Freeman M., Rohrer L., Zabrecky J., Matsudaira P., Krieger M. (1990). Type I macrophage scavenger receptor contains alpha-helical and collagen-like coiled coils. *Nature*.

[22] Endemann G., Stanton L. W., Madden K. S., Bryant C. M., White R. T., Protter A. A. (1993). CD36 is a receptor for oxidized low density lipoprotein. *The Journal of Biological Chemistry*.

[23] Sawamura T., Kume N., Aoyama T. (1997). An endothelial receptor for oxidized low-density lipoprotein. *Nature*.

[24] Martín-Fuentes P., Civeira F., Recalde D. (2007). Individual variation of scavenger receptor expression in human macrophages with oxidized low-density lipoprotein is associated with a differential inflammatory response. *Journal of Immunology*.

[25] Kunjathoor V. V., Febbraio M., Podrez E. A. (2002). Scavenger receptors class A-I/II and CD36 are the principal receptors responsible for the uptake of modified low density lipoprotein leading to lipid loading in macrophages. *The Journal of Biological Chemistry*.

[26] Rinne P., Rami M., Nuutinen S. (2017). Melanocortin 1 receptor signaling regulates cholesterol transport in macrophages. *Circulation*.

[27] Park Y. M. (2014). CD36, a scavenger receptor implicated in atherosclerosis. *Experimental & Molecular Medicine*.

[28] Febbraio M., Podrez E. A., Smith J. D. (2000). Targeted disruption of the class B scavenger receptor CD36 protects against atherosclerotic lesion development in mice. *The Journal of Clinical Investigation*.

[29] Kataoka H., Kume N., Miyamoto S. (1999). Expression of lectinlike oxidized low-density lipoprotein receptor-1 in human atherosclerotic lesions. *Circulation*.

[30] Morawietz H., Erbs S., Holtz J. (2006). Endothelial protection, AT1 blockade and cholesterol-dependent oxidative stress: the EPAS trial. *Circulation*.

[31] Zhou J., Febbraio M., Wada T. (2008). Hepatic fatty acid transporter Cd36 is a common target of LXR, PXR, and PPAR*γ* in promoting steatosis. *Gastroenterology*.

[32] Moore K. J., Rosen E. D., Fitzgerald M. L. (2001). The role of PPAR-gamma in macrophage differentiation and cholesterol uptake. *Nature Medicine*.

[33] Rios F. J., Jancar S., Melo I. B., Ketelhuth D. F. J., Gidlund M. (2008). Role of PPAR-gamma in the modulation of CD36 and FcgammaRII induced by LDL with low and high degrees of oxidation during the differentiation of the monocytic THP-1 cell line. *Cellular Physiology and Biochemistry*.

[34] Kim M. J., Lee Y. J., Yoon Y. S. (2018). A STAT6 inhibitor AS1517499 reduces preventive effects of apoptotic cell instillation on bleomycin-induced lung fibrosis by suppressing PPAR*γ*. *Cellular Physiology and Biochemistry*.

