# Supplementary material for: Circulating Levels of CILP2 Are Elevated in Coronary Heart Disease and Associated with Atherosclerosis
Source: Oxid Med Cell Longev. 2020 Oct 31;2020:1871984. doi: 10.1155/2020/1871984 (PMC7652603; doi:10.1155/2020/1871984)
Supplement: Supplementary Materials — See Supplementary Figures 1-5 and Supplementary Tables 1–3 in the Supplementary Material for comprehensive image analysis. Supplementary Table 1: the specific sequences used for CD36 promoter recombinant plasmids and site-directed mutation. Supplementary Table 2: the specific primers sequence used for RT-qPCR analysis. Supplementary Table 3: row mean scores and Cochran–Armitage trend test of the impact of circulating CILP2 on CHD patients. Supplementary Figure 1: flow of the study. Supplementary Figure 2: circulating CILP2 levels in SD-fed WT and ApoE KO mice (n = 6 for each group). Data are shown as mean ± SD. ∗∗p < 0.01 vs.C57BL/6J. Supplementary Figure 3: circulating CILP2 levels in SD- or HFD-fed ApoE KO mice. Eight-week-old male ApoE KO mice were divided into two groups (n = 6 for each group) and fed with a SD or HFD for 12 weeks. Data were shown as mean ± SD. ∗p < 0.05 vs. prefeeding. Supplementary Figure 4: the expression of CILP2 protein was found in the cultured THP-1 macrophages (A), THP-1 macrophages were transfected successfully by Ad-CILP2, and CILP2 expression was markedly evaluated at both mRNA and protein (B-D). Data are shown as mean ± SEM. ∗∗p < 0.01 vs. GFP. Supplementary Figure 5: cell apoptosis was determined by flow cytometry assay. Data were shown as mean ± SEM. ∗∗p < 0.01 vs. 0 h. [file 1871984.f1.docx]

**Supplementary materials**

**Circulating levels of CILP-2 are elevated in coronary heart disease and associated with atherosclerosis**

Wenjing Hu^1*^, Ke Li^2*^, Hongdong Han^2*^, Shan Geng^2^, Baoyong Zhou^3^, Xiaoyun Fan^1^, Shangcheng Xu^1^, Mengliu Yang^4^, Hua Liu^5^, Gangyi Yang^1^, Yongsheng Liu^1^

^1^Chongqing Prevention and Treatment Hospital for OccupationalDiseases,400000, Chongqing, China

^2^Department of Endocrinology, the Second Affiliated Hospital ,400000, Chongqing Medical University, Chongqing, China.

^3^Department of Hepatobiliary Surgery, First Affiliated Hospital, 400000,Chongqing Medical University, Chongqing, China

^4^School of Biomedical Sciences, the University of Queensland, 4702, Brisbane, Australia.

^5^Department of Pediatrics, University of Mississippi Medical Center, 32099, 2500 North State Street, 39200, Jackson, Mississippi, MS 39216-4505, USA

Wenjing Hu: 273297582@qq.com; Ke Li: [791450945@qq.com](mailto:791450945@qq.com); Hongdong Han:519699817@qq.com; Shan Geng: 83612914@qq.com; Baoyong Zhou: sdlylczhzh@163.com: Xiaoyun Fan: [188111010@qq.com](mailto:188111010@qq.com): Shangcheng Xu:xushangchengmito@163.com; Mengliu Yang: [yangmengliu@hotmail.com](mailto:184257881@qq.com); Hua Liu: hliu@umc.edu; Gangyi Yang: [gangyiyang@163.com](mailto:gangyiyang@163.com); Yongsheng Liu: [a273297582@126.com](mailto:a273297582@126.com);

**Running Head:** CILP-2 is associated with atherosclerosis.

*These authors contributed equally to this project

**Corresponding Author:** Gangyi Yang, E-mail: [gangyiyang@163.com](mailto:gangyiyang@163.com). and Yongsheng Liu, Chongqing Prevention and Treatment Hospital for Occupational Diseases, Chongqing, China. E-mail: [273297582@qq.com](mailto:273297582@qq.com) , +86-23-68485216 / Fax: +86-23-68485005.

Category: Original Article

**Word count of the abstract:** 243

**Word count of the main text:** 3933

**Table: *2* Figures:** 5 **References:** 34

**Clinical Trial Registration Number:** ChiCTR-OPC-14005324 **Table S1** The specific sequences used for CD36 promoter recombinant plasmids and site-directed mutation

| Promoter region | Primer |
| --- | --- |
| -404/187 | F: CCGCTCGAGCGGCGCAAGCTCAGTCAAGACAGGGAAGTG |
|  | R: CCCAAGCTTGGGCAGTAGTGTCACCTCCCGTCATCTGGGTGATG |
| -311/+187 | F: CCGCTCGAGCGGGTTAGGGGAAACTCAGCAAGTCAGTTC |
|  | R: CCCAAGCTTGGGCAGTAGTGTCACCTCCCGTCATCTGGGTGATG |
| -225/+187 | F: CCGCTCGAGCGGGCTGAATATCTCAGATATAGGTAATGGGTC |
|  | R: CCCAAGCTTGGGCAGTAGTGTCACCTCCCGTCATCTGGGTGATG |
| -102/187 | F: CCGCTCGAGCGGCCACACACTGGGATCTGACACTGTAG |
|  | R: CCCAAGCTTGGGCAGTAGTGTCACCTCCCGTCATCTGGGTGATG |
| mut PPRE-G | F: GTCAGTTCCTTTTGTTTTTCCAGCTTCTAACTTACTTGGATGGG |
|  | R: CCCATCCAAGTAAGTTAGAAGCTGGAAAAACAAAAGGAACTGAC |
| mut PPRE-J | F:GACCCTTATTAGCCATATCAGGCATGTACTGTATGGGGGATTTTTTTT |
|  | R: AAAAAAAATCCCCCATACAGTACATGCCTGATATGGCTAATAAGGGTC |

**Table S2** The specific primers sequence used for RT-PCR analysis

| Gene | Forward and reverse primers | Amplified fragment (bp) | Annealing  temperature (ºC) |
| --- | --- | --- | --- |
| β-actin | 5'-GCTGTCCCTGTATGCCTCT-3' | 220 | 55 |
|  | 5'-GATGTCACGCACGATTTCC-3' |  |  |
| CILP2 | 5’-GTGTGGTATGGAATGGAGTT-3’ | 119 | 55 |
|  | 5’-TACGCACGAGAAGTCAGA-3’ |  |  |
| cilp2 | 5’- AGACTCAGGCCTTAGGTGGA -3’ | 86 | 62.5 |
|  | 5’- GTTTGCAGTTCTCATCCCTCT -3’ |  |  |
| SR-A | 5’-CACCTTGACACTACACCCTT-3’ | 142 | 50 |
|  | 5’- TTCCTCTTCGCTGTCATTTC -3’ |  |  |
| CD36 | 5’- TAACCCAGGACGCTGAGGA-3’ | 115 | 50 |
|  | 5’-GCCAGATTGAGAACTGTGAAG-3’’ -3’ |  |  |
| LOX-1 | 5’- CCCTCAAAGTGTCACAATGCT -3’ | 140 | 50 |
|  | 5’-GCAAAGTGAAAGAAGCCAGAC-3’ -3’ |  |  |

**Table S3** Row Mean Scores and Cochran–Armitage Trend test of the impact of circulating CILP2 on CHD patients

|  |  | CAD | |  |
| --- | --- | --- | --- | --- |
| Model adjusted |  | X^2^ | *p* |  |
| Row Mean Scores Test |  | 45.1422 | < 0.0001 |  |
| Cochran–Armitage Trend Test |  | -4.7658 | < 0.0001 |  |

**Analysis**

Analysed (n = 105)

Excluded from analysis (n = 0)

Allocated to intervention (CHD = 170)

Received allocated intervention (n = 169)

Did not receive allocated intervention (n = 1)

Excluded (n = 61)

-Not meeting inclusion criteria (n = 51)

-Declined to participate (n = 10)

-Other reasons (n = 0)

**Enrollment**

Allocated to intervention (NS = 110)

Received allocated intervention (n = 105)

Did not receive allocated intervention (n= 5)

Assessed for eligibility (CHD > 150, NS > 150)

**Allocation**

Analysed (n=167)

Excluded from analysis (n = 2)

**Figure S1** Flow of the study


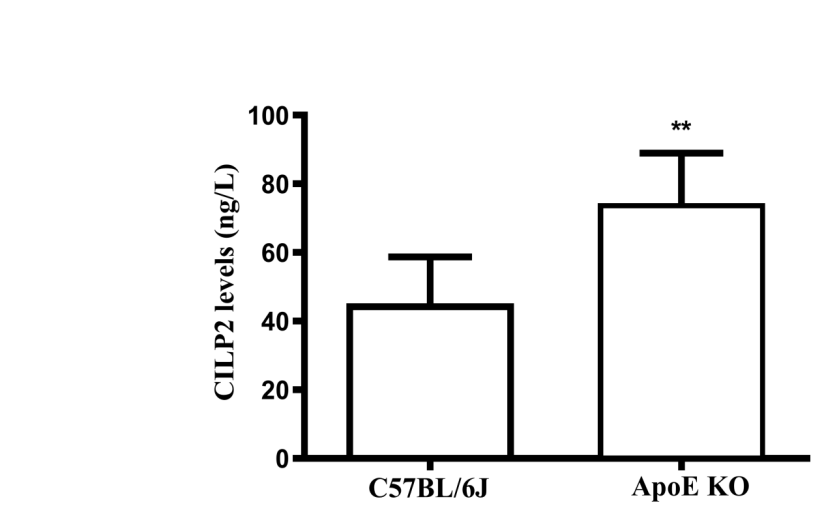


**Figure S2** Circulating CILP2 levels in SD-fed WT and ApoE KO mice (n = 6 for each group). Data were shown as mean ± SD. ***p* < 0.01 *vs.* C57BL/6J.


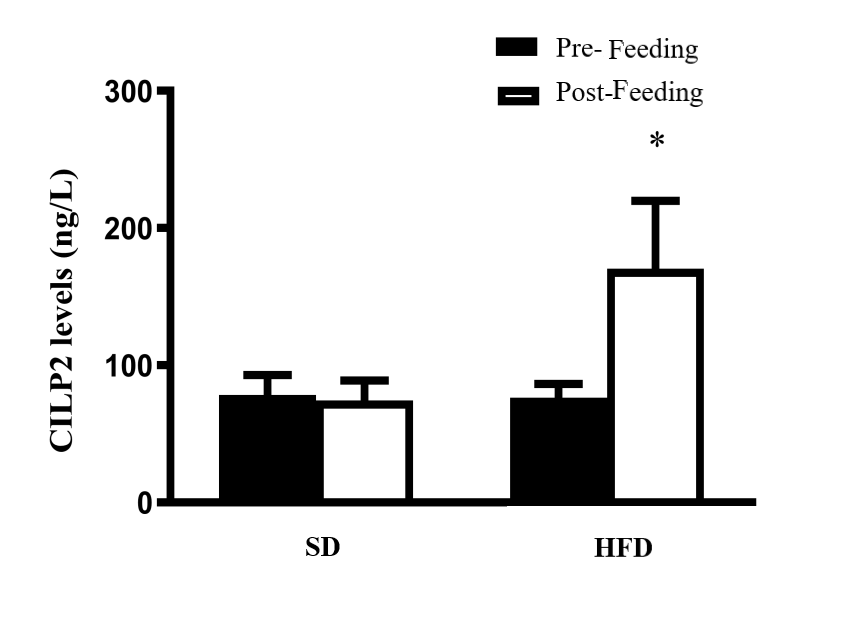


**Figure S3** Circulating CILP2 levels in SD- or HFD-fed ApoE KO mice. Eight-week-old male ApoE KO mice were divided into two groups (n = 6 for each group) and fed with a SD or HFD for 12 weeks. Data were shown as mean ± SD. * *p* < 0.05 *vs.* pre-feeding.


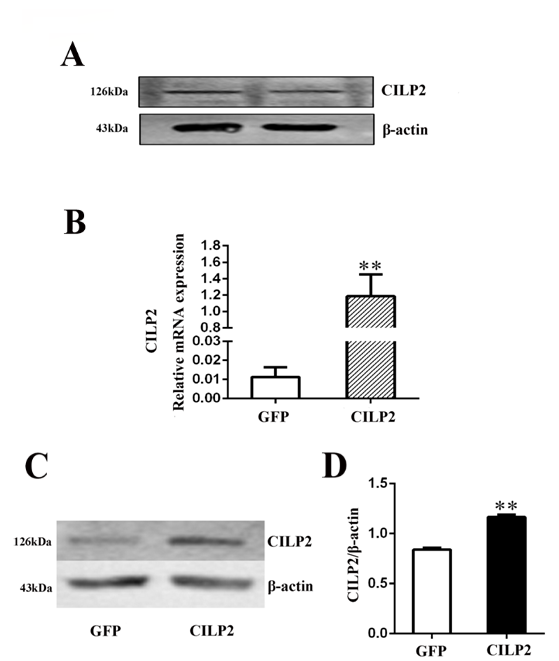


**Figure S4** The expression of CILP2 protein was found in the cultured THP-1 macrophages (A), THP-1 macrophages were transfected successfully by Ad-*CILP2*, and CILP2 expression was markedly evaluated at both mRNA and protein (B-D). Data are shown as mean ± SEM. ^**^*p* < 0.01 *vs.* GFP.


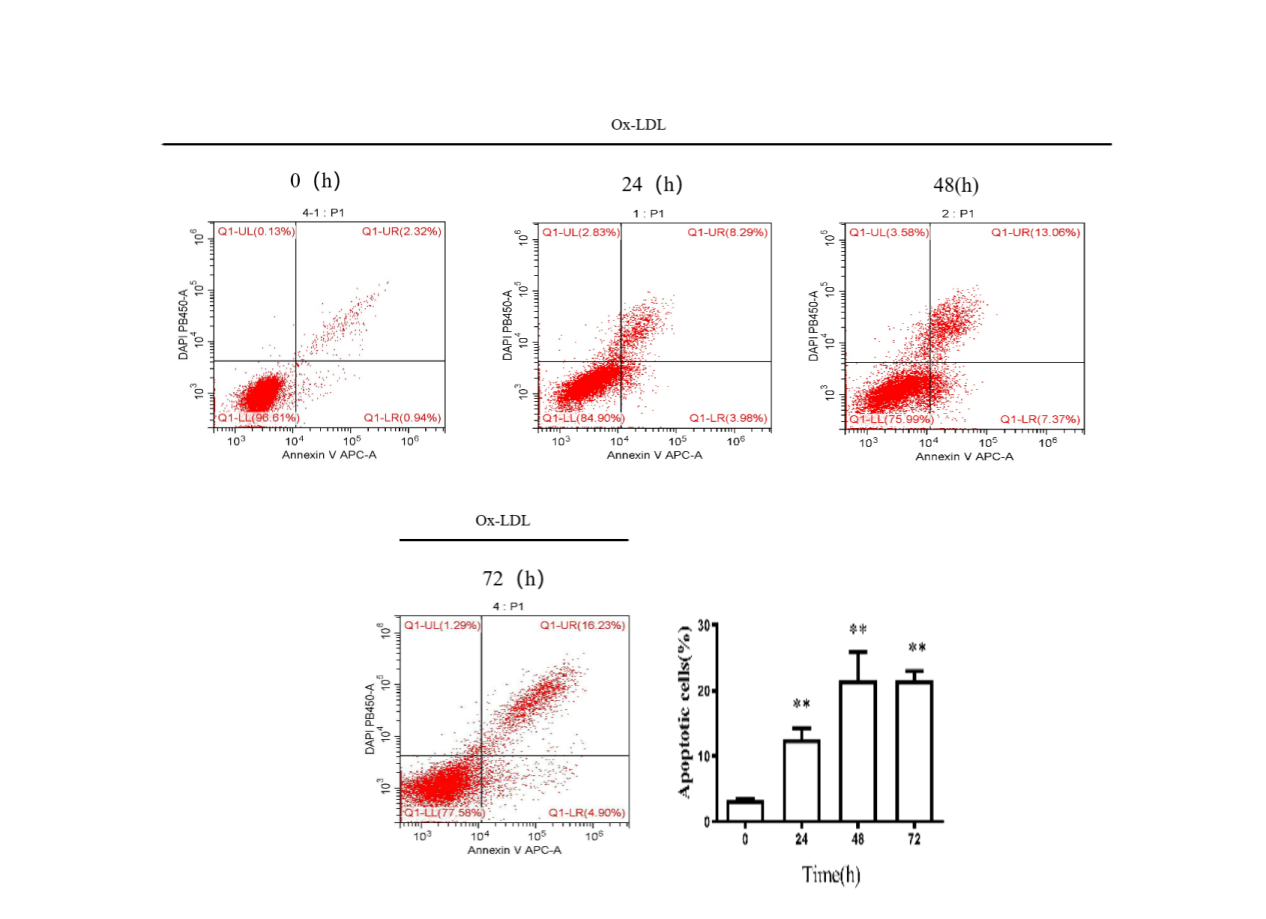


**Figure S5** Cell apoptosis was determined by flow cytometry assay. Data were shown as mean ± SEM. ^**^*p* < 0.01 *vs.* 0 h.
